# Establishment of epidemiological cutoff values for yeast-phase *Talaromyces marneffei* against six antifungal agents: a multicenter study in China

**DOI:** 10.1128/aac.00650-26

**Published:** 2026-08-05

**Authors:** Kai-su Pan, Ying-gai Song, Sha Lu, Xiao-fang Li, Huai-qiu Huang, Jie Gong, Ruo-yu Li, Hai-lin Zheng, Miao-rong Zhan, Shu Zhang, Xin-yu Zhou, Yuan-yuan Geng, Si-wen Jiang, Sheng-xiu Fang, Yanqing Zheng, Dong-yan Zheng, Weixuan Wu, Li-yan Xi, He-ping Xu, Paul E. Verweij, Cun-wei Cao

**Affiliations:** 1 Department of Dermatology, The First Affiliated Hospital of Guangxi Medical University 117742, Nanning, China; 2 Guangxi Scientific and Technological Innovation Cooperation Base of Mycosis Prevention and Control, Nanning, China; 3 Department of Dermatology and Venerology, Peking University First Hospital 26447, Beijing, China; 4 Department of Dermatology, Sun Yat-sen Memorial Hospital, Sun Yat-sen University 622951, Guangzhou, China; 5 Department of Medical Mycology, Institute of Dermatology and Hospital for Skin Diseases, Chinese Academy of Medical Sciences & Peking Union Medical College 70575, Nanjing, China; 6 Department of Dermatology and Venereology, The Third Affiliated Hospital of Sun Yat-Sen University, Guangzhou, China; 7 National Key Laboratory of Intelligent Tracking and Forecasting for Infectious Diseases, National Institute for Communicable Disease Control and Prevention, Chinese Center for Disease Control and Prevention 12415, Beijing, China; 8 The First Affiliated Hospital of Guilin Medical University, Guilin, China; 9 The Fourth People’s Hospital of Nanning, Nanning, China; 10 Fangchenggang Wanqing Institute of Mycosis Prevention and Control, Fangchenggang, China; 11 Dermatology Hospital of Southern Medical University, Guangzhou, China; 12 Department of Laboratory Medicine, The First Aﬃliated Hospital of Xiamen University, Xiamen, Fujian, China; 13 Centre for Infectious Diseases Research, Diagnostics and Laboratory Surveillance, National Institute for Public Health and the Environment (RIVM) 10206, Bilthoven, the Netherlands; 14 Department of Medical Microbiology and Radboudumc - CWZ Centre of Expertise for Mycology, Radboud University Medical Centre, Nijmegen, the Netherlands; University of Iowa, Iowa City, Iowa, USA

**Keywords:** *Talaromyces marneffei*, epidemiological cutoff value, antifungal susceptibility test, acquired drug resistance, wild-type, ECOFFinder

## Abstract

This study aimed to establish epidemiological cutoff values (ECVs) for amphotericin B, fluconazole, itraconazole, voriconazole, posaconazole, and isavuconazole against *Talaromyces marneffei* (*T. marneffei*) using the CLSI broth microdilution method. Antifungal susceptibility testing of 207 *T. marneffei* isolates was performed independently at five laboratories across China according to CLSI M27-A4 guidelines. Minimum inhibitory concentration (MIC) distributions were analyzed using ECOFFinder software (version 2.1), and ECVs were set at the upper MIC limit encompassing 97.5% of the modeled wild-type (WT) population. ECVs were determined as follows: amphotericin B (AMB) 8 µg/mL, voriconazole (VRC) 0.03 µg/mL, itraconazole (ITC) 0.125 µg/mL, posaconazole (POS) 0.06 µg/mL, isavuconazole (ISA) 0.125 µg/mL, and fluconazole (FLC) 8 µg/mL. MIC distributions for all six agents conformed well to the fitted models (normal or log-normal), supporting the validity of ECV determination. No non-WT isolates were detected for amphotericin B, whereas non-WT rates for azoles were 15.5% (voriconazole), 8.45% (itraconazole), 6.37% (posaconazole), 3.0% (fluconazole), and 1.02% (isavuconazole). Among voriconazole non-WT isolates, all were from patients with prior voriconazole exposure. This study provides the first standardized ECVs for *T. marneffei* against six key antifungal agents. The data establish a reference framework for antifungal susceptibility testing and resistance surveillance and highlight the need for continued monitoring of azole susceptibility in this pathogen.

## INTRODUCTION

Talaromycosis is an invasive fungal disease caused by the dimorphic fungi *Talaromyces marneffei* (*T. marneffei*). Since *T. marneffe* was first isolated from the liver of Vietnamese bamboo rats in 1956 (1) and the first human case was reported in 1973 (2), talaromycosis has long been recognized as a major opportunistic infection in severely immunocompromised individuals, particularly those with advanced HIV/AIDS, in whom it causes significant morbidity and mortality (3). With the expansion of its geographical range and the increasing number of non-HIV immunocompromised patients (e.g., those with autoimmune diseases, transplant recipients, or patients on immunosuppressive therapies) being diagnosed, the clinical burden of this infection is evolving, highlighting the increasing global health importance of this neglected mycosis (4).

At present, the first-line treatment of talaromycosis includes amphotericin B (AMB) (induction phase) and itraconazole (ITC) (consolidation and maintenance phase). Voriconazole (VRC) is often used in the induction phase for patients who cannot tolerate amphotericin B, and posaconazole (POS) and isavuconazole (ISA) have also been shown to have good efficacy (5–7). However, prolonged, extensive, and even inappropriate azole exposure, particularly for secondary prevention, creates strong selection pressure that could theoretically lead to the emergence of acquired resistance. Although cases of clinical treatment failure have occasionally been reported (8, 9), due to the lack of clinical breakpoints, epidemiological cutoff values (ECV or ECOFF) can be used to distinguish between wild-type (WT) strains (without acquired resistance mechanisms) and non-WT strains (which may carry acquired resistance mechanisms). Estimates of the frequency of non-WT isolates are important for surveillance and guidance of local treatment recommendations.

Currently, there are no ECVs available for *T. marneffei* utilizing the Clinical and Laboratory Standards Institute (CLSI) and European Committee on Antimicrobial Susceptibility Testing (EUCAST) reference methods. Clinically, in patients with treatment failure or relapse, it is often difficult to determine if antifungal resistance plays a role in addition to host factors and antifungal drug exposure. Therefore, the establishment of a reliable ECV system is not only an urgent need for laboratory standardization but also a prerequisite for precision antifungal treatment, effective drug resistance surveillance, and public health early warning.

Establishing ECVs requires at least 100 isolates and *in vitro* susceptibility testing in multiple laboratories. As *T. marneffei* is restricted geographically, there is overall limited experience in susceptibility testing of this fungus. China, as the main epidemic area of *T. marneffei*, has large clinical strain resources, which provides a unique opportunity to determine ECVs. This study aims to establish ECV data using the CLSI reference method by analyzing isolates collected from different host sources. These data provide an important foundation for future antifungal susceptibility interpretation and resistance surveillance of *T. marneffei*.

## MATERIALS AND METHODS

### Source of strains and inclusion criteria

The strains in this study were obtained from the collections of five participating laboratories (the First Affiliated Hospital of Guangxi Medical University, the First Affiliated Hospital of Xiamen University, Peking University First Hospital/Chinese Center for Disease Control and Prevention, Hospital of Dermatology, Chinese Academy of Medical Sciences, and the Third Affiliated Hospital of Sun Yat-sen University). The inclusion criteria were as follows: (i) *T. marneffei* confirmed by morphology and ITS region sequencing; (ii) clinical isolates were recovered from patients with confirmed HIV status or non-HIV immunocompromised patients (including those with anti-IFN-γ autoantibody positivity, malignant tumor, non-tuberculous mycobacterial infection, abnormal liver and kidney function, or other immunocompromised conditions); clinical specimens included blood, bone marrow, sputum, skin tissue, and other sterile or non-sterile site specimens; environmental isolates were obtained from bamboo rat viscera or soil (detailed host and underlying disease distributions are provided in Table S1); (iii) non-repetitive strains from different patients or sources; (iv) the inclusion period was from January 2018 to June 2025.

### Strain resuscitation and identification

All strains were revived at −80°C before experiments, inoculated on Sabouraud dextrose agar (SDA) plates, and cultured at 25°C until the typical hyphal phase (producing wine red pigment). Morphological confirmation was performed by microscopic observation of the broom branch structure. At the same time, genomic DNA was extracted by the CTAB method, and the universal primers ITS1/ITS4 were used to amplify the ITS rDNA region, and sequencing was performed. The sequence was compared with the standard sequence in the CBS database to complete molecular biological identification.

### *In vitro* antifungal susceptibility test

The Reference method for Antifungal susceptibility Testing by broth microdilution Method for Yeast cells (M27-A4) issued by the CLSI (10) was used and slightly modified according to the slow growth characteristics of strains, mainly as the inoculation concentration was adjusted from 2 to 5 × 10^3^ to 2–5 × 10⁴, and the minimum inhibitory concentration (MIC) was read at 72 h of incubation, in accordance with previous publications (11–15).

For yeast cell suspension preparation, pure cultures were inoculated on brain heart infusion (BHI) broth and incubated at 37°C for 3–10 days, followed by daily microscopic observation. The yeast cells were collected when more than 95% of the cells had changed to the typical yeast phase (dachshund shape with transverse septum). The yeast cell suspension concentration was adjusted to 2–5 × 10⁶ colony-forming unit (CFU)/mL using a hemocytometer and then diluted 100 times with RPMI-1640 medium (MOPS buffer, pH 7.0) to obtain a 2× working yeast suspension with a final concentration of 2–5 × 10⁴ CFU/mL.

Antifungal drugs included amphotericin B (AMB), fluconazole (FLC), itraconazole (ITC), voriconazole (VRC), posaconazole (POS), and isavuconazole (ISA). Drug powder was purchased from Sigma-Aldrich Co. High-concentration storage solutions (at least 10 times the highest tested concentration) were prepared using dimethyl sulfoxide (DMSO) and stored in aliquots at −80°C. A 96-well microtiter plate was used to prepare the drug solution in 10 concentration gradients by serial two-fold dilutions of the stock solution using RPMI-1640 medium. The drug concentration range was set as AMB 16–0.031 μg/mL; for FLC, it was 64–0.125 μg/mL; for ITC, VRC, POS, and ISA, the drug concentration range was 4–0.008 μg/mL. A volume of 100 μL of the diluted yeast suspension and 100 μL of the working drug solution were added to each well, resulting in a final inoculum of 1–2.5 × 10⁴ CFU/mL, where the drug concentration is the default gradient. Growth control (only yeast inoculum) and blank control (only culture medium) were set up in each plate. After capping, the plates were incubated at 37°C for 72 h. After 72 h of incubation, the absorbance (OD) of each well was read at a wavelength of 530 nm using a microplate reader, and the plates were also read visually. The following MIC endpoints were used: for AMB, the lowest drug concentration that caused 100% growth inhibition; for FLC, ITC, VRC, POS, and ISA, the lowest drug concentration that caused 50% growth inhibition. All susceptibility plate results were photographed for archiving. For each plate, we determined the MIC endpoint primarily by visual reading according to the CLSI M27-A4 standard, as described above. The OD value at 530 nm was measured by a microplate reader as an objective reference. When the visual results did not agree with the OD value, the isolates were retested, and the final MICs were determined based on the retested results.

### Quality control

The quality control strains recommended by the CLSI were as follows: *Candida krusei* ATCC 6258 and *C. parapsilosis* ATCC 22019 were used for each assay. The batch test data were considered valid only if the MIC value of the quality control strain fell within the allowable range specified in the CLSI document M27-A4 (10).

### Data validity and sample size explanation

To ensure the accuracy and reproducibility of MIC readings, isolates with unclear growth endpoints or those failing quality control (e.g., out-of-range values for control strains) were excluded per CLSI M27-A4 guidelines. This led to a slight reduction in the number of valid isolates for some agents, particularly those with narrower MIC distributions or stricter endpoint criteria (e.g., azoles). Importantly, the final sample sizes for all drugs exceeded 100 isolates, which is substantially above the recommended threshold for ECV determination. To further verify the reliability and stability of MIC values, a subset of isolates was tested in multiple participating laboratories with overlapping coverage. For any strain tested in more than one laboratory, only one valid MIC data set was included in the final analysis to ensure data independence. Thus, the present study fulfills all three core CLSI M57 requirements for ECV establishment, including testing of more than 100 isolates, participation of at least three independent laboratories, and the absence of any single laboratory contributing more than 50% of the total data set.

### Data collection and statistical analysis

Each participating laboratory submitted raw MIC data (in μg/mL) to the central laboratory. Data from all laboratories were combined to form a total MIC data set for each drug. Statistical analyses were performed using ECOFFinder software (version 2.1) developed by Turnidge et al. and recommended by CLSI (16). The software fitted the MIC distribution data by the maximum likelihood method, established a normal or lognormal distribution model, and calculated the MIC upper limit values covering different proportions of wild-type strains (e.g., 95%, 97.5%, and 99%). According to CLSI M57 guidelines, the upper limit usually chooses 97.5% of wild-type subset MIC as the ultimate ECV (16–19).

## RESULTS

### MIC distributions and establishment of epidemiological cutoff values (ECVs)

A total of 207 isolates of *T. marneffei* were included in the antifungal susceptibility analysis. After rigorous quality control, effective isolates were obtained, with the specific numbers as follows: 196 isolates for amphotericin B (AMB), 203 isolates for fluconazole (FLC), 189 isolates for itraconazole (ITC), 200 isolates for voriconazole (VRC), 204 isolates for posaconazole (POS), and 196 isolates for isavuconazole (ISA) (Table 1). All effective sample sizes exceeded 100 isolates, meeting the requirements of the CLSI for ECV, ensuring the reliability of statistical analysis.

**TABLE 1:** MIC distributions and ECVs for six antifungal agents against *T. marneffei*^*a*^

Antifungal agent	Effective number of strains	MIC range (μg/mL)	MIC₅₀ (μg/mL)	ECV (97.5%, μg/mL)	Non-WT isolates, n (%)
Amphotericin B	196	0.25–8	1	8	0 (0.0)
Fluconazole	203	0.25–64	2	8	6 (3.0)
Itraconazole	189	0.008–0.5	0.03	0.125	16 (8.45)
Voriconazole	200	0.008–0.5	0.015	0.03	31 (15.5)
Posaconazole	204	0.008–0.25	0.015	0.06	13 (6.37)
Isavuconazole	196	0.002–0.25	0.015	0.125	2 (1.02)

^*a*^ MIC values were determined by CLSI broth microdilution. ECVs were established according to CLSI M57 guidelines using ECOFFinder software and represent the MIC encompassing ≥97.5% of the modeled wild-type population. The MIC₅₀ was determined as the MIC values at the 50th percentile. ECVs were determined using ECOFFinder based on the 97.5% wild-type population according to CLSI M57 guidelines. Non-WT isolates were defined as isolates with MIC values above the ECV.

**Fig 1:**
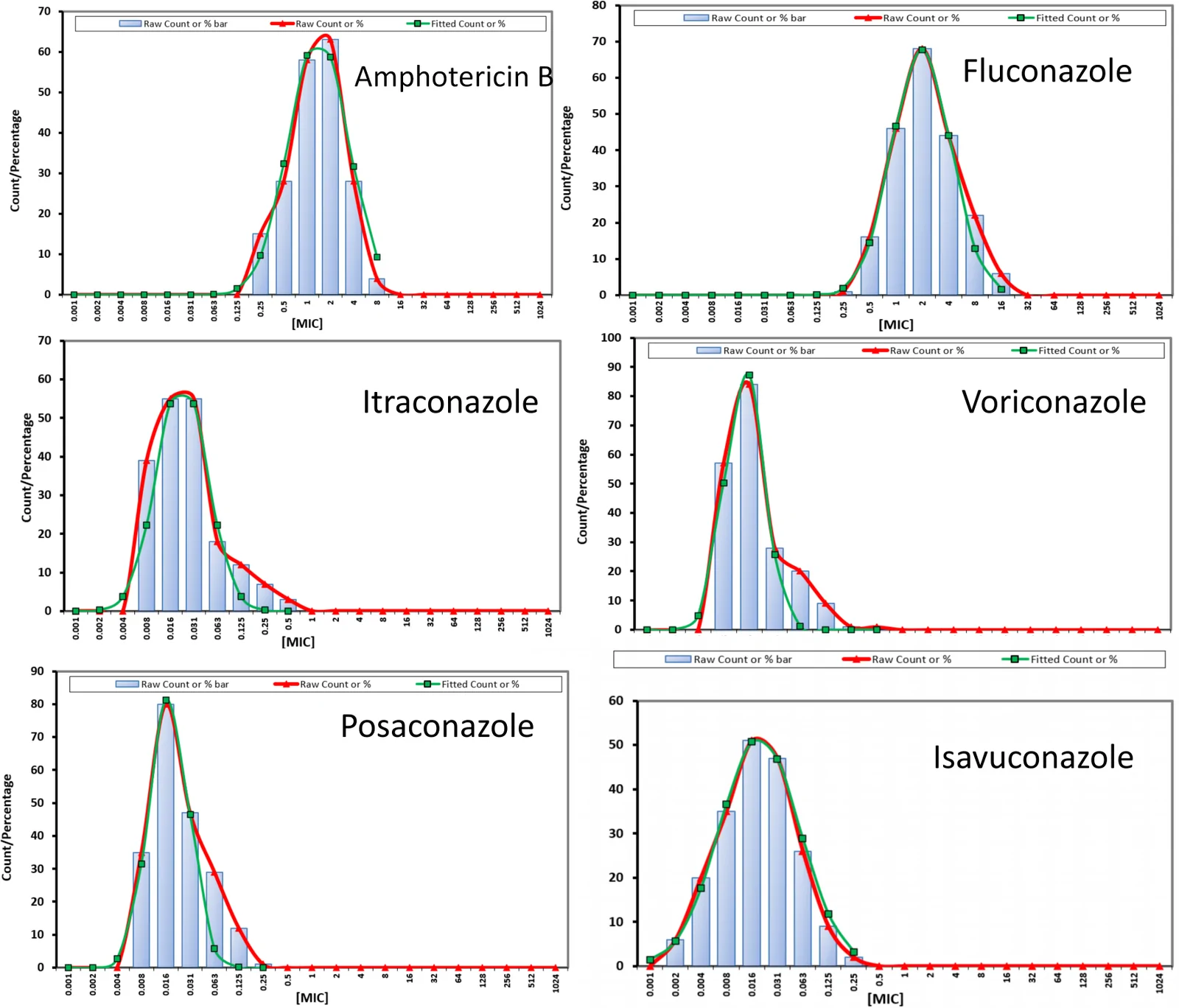
MIC distribution profiles of six antifungal agents against *T. marneffei*. Histograms represent the observed MIC frequency distributions for amphotericin B, fluconazole, itraconazole, voriconazole, posaconazole, and isavuconazole. Red and green curves indicate the raw and modeled (fitted) distributions used for ECV determination according to CLSI M57 guidelines.

The ECVs (97.5% wild-type) for the six antifungal agents were successfully determined: AMB 8 μg/mL, FLC 8 μg/mL, ITC 0.125 μg/mL, VRC 0.03 μg/mL, POS 0.06 μg/mL, and ISA 0.125 μg/mL (Table 1). The MIC fitting curve indicated that the MIC data for each drug conformed to the WT normal distribution or lognormal distribution, with excellent model fit (Fig. 1). The MIC frequency distributions of the six drugs exhibited different characteristics, with AMB concentrated in the range of 0.25–8 μg/mL, while azole drugs were mainly distributed in the low concentration range of 0.008–0.25 μg/mL (Table 2). The MIC_50_ values of each antifungal agent were also calculated to reflect the overall susceptibility profile of *T. marneffei*. MIC distributions across participating centers are presented in Tables S2 to S7.

**TABLE 2:** The frequency distribution of MICs of six antifungal drugs against *T. marneffei*

	0.002	0.004	0.008	0.015	0.03	0.06	0.125	0.25	0.5	1	2	4	8	16	32	64
Amphotericin B	–^*a*^	–	–	–	–	–	–	15	28	58	63	28	4	–	–	–
Fluconazole	–	–	–	–	–	–	–	1	16	46	68	44	22	6	–	–
Itraconazole	–	–	39	55	55	18	12	7	3	–	–	–	–	–	–	–
Voriconazole	–	–	57	84	28	20	9	1	1	–	–	–	–	–	–	–
Posaconazole	–	–	35	80	47	29	12	1	–	–	–	–	–	–	–	–
Isavuconazole	6	20	35	51	47	26	9	2	–	–	–	–	–	–	–	–

^*a*^ –, MIC concentrations not tested for the specific agent or with zero observed isolates.

### Identification of non-WT isolates

Based on the established ECVs, non-wild-type (NWT) isolates (MIC >ECV) were identified for each antifungal agent, with significant differences in the proportion of NWT isolates among drugs (Table 1). No NWT isolates were found for AMB (0.0%, 0/196). The proportion of NWT isolates for triazole drugs was relatively notable, with POS (6.37%, 13/204), ITC (8.45%, 16/189), and VRC (15.5%, 31/200) showing the highest ratios. The proportions of NWT isolates for FLC and ISA were 3.0% (6/203) and 1.02% (2/196), respectively. Among the 31 non-wild-type strains of voriconazole, all were recovered from patients with documented prior voriconazole exposure.

### Demographic and underlying disease distribution of isolates

A summary of isolate sources and patient demographics is provided in Table S1. Of the 207 isolates included in the study, 187 (90.3%) were clinical isolates and 20 (9.7%) were environmental isolates. Among the clinical isolates, 123 were obtained from HIV-positive patients and 64 from non-HIV immunocompromised patients, among whom anti-IFN-γ autoantibody positivity was the most common underlying condition. Detailed demographic characteristics, underlying diseases, and MIC distributions across participating centers are provided in Tables S1 to S7.

## DISCUSSION

The ECVs of *T. marneffei* to six key antifungal agents were successfully established in this study, representing an essential step toward standardized antifungal susceptibility testing and resistance surveillance for this pathogen. For a long time, there has been no unified standard for the interpretation of drug susceptibility results of *T. marneffei*. Clinicians and microbiologists can only refer to MIC values, limited epidemiological data, or *Candida* breakpoints for rough evaluation, which is methodologically unsound and may lead to inaccurate interpretation. The ECVs determined in this study provide, for the first time, statistically defined thresholds that allow laboratories to distinguish wild-type from non-wild-type populations based on objective population-level data, thereby reducing interpretative uncertainty and improving comparability across studies. Importantly, the study fulfilled all CLSI M57 requirements for ECV determination, including testing of more than 100 isolates, participation of multiple independent laboratories, and balanced laboratory contributions. Therefore, the ECVs generated in this study can be considered methodologically robust and suitable for use in future surveillance studies.

For AMB, the ECV was determined to be 8 µg/mL, with no NWT isolates found among 196 valid isolates, which is consistent with previous reports indicating that *T. marneffei* MIC distributions for AMB remain within the wild-type range, with no non-wild-type isolates detected (20). The highly consistent MIC distribution of AMB across participating centers supports the stability of its wild-type MIC range. Notably, the ECV of AMB for *T. marneffe*i underscores the necessity of species-specific susceptibility interpretation and highlights the limitations of directly applying breakpoints derived from other fungal pathogens.

In this study, antifungal susceptibility testing was performed exclusively on the yeast phase of *T. marneffei*. This approach was chosen because the yeast form represents the pathogenic morphology observed in human tissue, and the CLSI M27-A4 broth microdilution method is specifically standardized for yeast-form cells. Testing the yeast phase, therefore, provides clinically relevant MIC data that reflect the form of the fungus encountered by the host during infection. Whether MIC values obtained from yeast-phase and mycelial-phase susceptibility testing correlate with one another remains unclear and warrants further investigation. Furthermore, although the CLSI M38 reference method provides a standardized methodology for susceptibility testing of filamentous fungi, ECVs derived from mycelial-phase testing of *T. marneffei* have not yet been established.

The elevated proportion of non-wild-type isolates (15.5%) observed for voriconazole—the highest among all six agents tested—warrants particular attention. Notably, all 31 voriconazole non-wild-type isolates in this study were recovered from patients who had received voriconazole as part of their induction or maintenance therapy, a clinical context that provides a plausible basis for azole selection pressure (21, 22). However, the observation that most patients receiving voriconazole therapy still harbored wild-type isolates with low MICs indicates that drug exposure alone may not fully explain the emergence of elevated MICs. This observation may suggest that host factors, duration and intensity of drug exposure, or strain-specific genetic predispositions (e.g., pre-existing or acquired cyp51 gene alterations) may collectively contribute to the development of reduced susceptibility (23, 24). A recent study has suggested potential cross-resistance mechanisms involving cyp51B in *T. marneffei* exposed to voriconazole (24). Furthermore, the non-wild-type rates for itraconazole (8.45%) and posaconazole (6.37%) further underscore the need for vigilance across the azole class as cross-resistance among these compounds remains a concern (23, 24). While the MIC_50_ of voriconazole remained low (0.015 µg/mL), the clinical relevance of these non-wild-type phenotypes requires a prospective correlation with treatment outcomes. Continued molecular characterization of non-wild-type isolates, particularly those from patients with documented prolonged azole exposure, is urgently needed.

FLC displayed a high ECV (8 µg/mL), which is consistent with previous observations that fluconazole has limited activity against *T. marneffei* and is not recommended as a first-line treatment option. Nevertheless, the ECV established in this study provides a useful framework for identifying isolates with unusually high MICs, which may serve as a starting point for investigating intrinsic resistance mechanisms and monitoring long-term susceptibility trends.

The demographic profile of the isolate collection is consistent with the evolving epidemiology of talaromycosis. Although HIV-positive patients remained the predominant source of clinical isolates, a considerable proportion of isolates were recovered from non-HIV immunocompromised individuals, particularly those with anti-IFN-γ autoantibodies. These findings reinforce the growing recognition of talaromycosis as an important opportunistic infection beyond the HIV population and underscore the need for increased clinical awareness in this expanding at-risk group.

Previous studies have reported MIC distributions for *T. marneffei* isolates (11–13, 25); however, these investigations were limited by small sample sizes, heterogeneous methodologies, and the absence of statistically defined ECVs derived according to standardized criteria. In contrast, the present study represents the first comprehensive ECV determination conducted in strict accordance with CLSI M57 guidelines, using ECOFFinder and a large, multicenter isolate collection. The resulting ECV data set may serve as an essential first step toward future clinical breakpoint development for *T. marneffei* through integration with pharmacokinetic-pharmacodynamic (PK/PD) and clinical outcome data.

This study also has several limitations. First, the strains were mainly collected from China, and the applicability of the ECVs in other endemic areas (e.g., Southeast Asian countries) needs further verification. Second, ECV is a microbiological parameter and does not directly correlate with clinical outcomes; the establishment of formal clinical breakpoints requires the integration of pharmacokinetic-pharmacodynamic (PK/PD) data and clinical response analyses. Third, the molecular mechanisms underlying the NWT phenotypes were not investigated in this study, and the precise genetic basis of reduced susceptibility to azoles (e.g., mutations in the cyp51 gene family) remains to be elucidated, although previous studies have characterized resistance mechanisms in fluconazole-resistant *T. marneffei* (26). Fourth, some patients had multiple underlying diseases, which may affect the analysis of the correlation between a single underlying disease and drug susceptibility, and further large-sample cohort studies are needed to explore this relationship. Finally, environmental isolates were included in the study, but their sample size was small (20 cases), and the drug susceptibility differences between clinical and environmental isolates need to be further studied with a larger sample size. Notably, a related study by our group found that clinical *T. marneffei* isolates exhibited diminished antifungal responsiveness and enhanced stress tolerance relative to non-clinical strains (27), suggesting that such comparisons may reveal biologically meaningful phenotypic differences.

### Conclusion

In conclusion, this study established standardized epidemiological cutoff values for AMB, FLC, ITC, VRC, POS, and ISA against *T. marneffei*. The ECVs generated provide a reliable and objective framework for laboratories to identify non-wild-type isolates and support epidemiological surveillance. No non-wild-type isolates were identified for amphotericin B in the present collection. In contrast, a noteworthy proportion of non-wild-type isolates was observed for certain second-generation triazoles, underscoring the need for continued monitoring and further mechanistic studies. Overall, this work provides the first CLSI-based ECV framework for *T. marneffei* and represents an important step toward standardized antifungal susceptibility interpretation, resistance surveillance, and future clinical breakpoint development for this emerging pathogen.

## Data Availability

All data generated or analyzed during this study are included in this published article and its Supplemental material.

## SUPPLEMENTAL MATERIAL

Supplemental material aac.00650-26-s0001.docx Tables S1 to S7.

ASM does not own the copyrights to Supplemental Material that may be linked to, or accessed through, an article. The authors have granted ASM a non-exclusive, world-wide license to publish the Supplemental Material files. Please contact the corresponding author directly for reuse.
