# Supplementary material for: Establishment of epidemiological cutoff values for yeast-phase Talaromyces marneffei against six antifungal agents: a multicenter study in China
Source: Antimicrob Agents Chemother. 2026 Aug 5;70(9):e00650-26. doi: 10.1128/aac.00650-26 (PMC13712858; doi:10.1128/aac.00650-26)
Supplement: Supplementary file 1 [file aac.00650-26-s0001.docx]

**Table S1. Demographic and Underlying Disease Distribution of *T. marneffei* Isolates.**

| **Classification Dimension** | **No. of Cases (n)** | **Proportion(%)** |
| --- | --- | --- |
| **I. Source of Isolates** | **207** | **100.0** |
| Isolates from HIV-positive patients | 123 | 59.42 |
| Isolates from non-HIV immunocompromised patients | 64 | 30.92 |
| Environmental isolates (from bamboo rat viscera, soil, etc.) | 20 | 9.66 |
| **II. Underlying Disease Subgroups of Non-HIV Immunocompromised Patients** |  |  |
| Positive for anti-IFN-γ autoantibodies | 50 | 78.125 |
| Complicated with malignant tumor | 8 | 12.5 |
| Complicated with non-tuberculous mycobacterial infection | 9 | 14.06 |
| Other immunocompromised conditions (abnormal liver and kidney function) | 15 | 30 |
| **III. Gender Distribution (clinical patient isolates only)** |  |  |
| Male | 87 | 42.03 |
| Female | 120 | 57.97 |
| **IV. Age Distribution (clinical patient isolates only, years)** |  |  |
| ≤18 years | 11 | 5.88 |
| 18–60 years | 131 | 70.05 |
| >60 years | 45 | 24.06 |
| Mean age (±SD) | 50.1±15.5 | — |

**Table S2. The MIC distribution of AMB in each center.**

| **Center** | **N** | **MIC range** | **MIC₅₀** | **MIC₉₀** |
| --- | --- | --- | --- | --- |
| **A** | 30 | 1-4 | 2 | 4 |
| **B** | 39 | 0.5-2 | 1 | 2 |
| **C** | 43 | 0.25-8 | 2 | 4 |
| **D** | 38 | 0.25-8 | 1 | 4 |
| **E** | 46 | 0.25-4 | 1 | 2 |
| **Total** | 196 | 0.25-8 | 1 | 4 |

**A.** Peking University First Hospital/Chinese Center for Disease Control and Prevention, **B.** the First Affiliated Hospital of Xiamen University, **C.** Hospital of Dermatology, Chinese Academy of Medical Sciences, **D.** the Third Affiliated Hospital of Sun Yat-sen University, **E.** the First Affiliated Hospital of Guangxi Medical University.

**Table	S3. The MIC distribution of FLC in each center.**

| **Center** | **N** | **MIC range** | **MIC₅₀** | **MIC₉₀** |
| --- | --- | --- | --- | --- |
| **A** | 46 | 0.5-4 | 2 | 4 |
| **B** | 40 | 1-32 | 2 | 8 |
| **C** | 42 | 1-16 | 2 | 8 |
| **D** | 35 | 0.5-16 | 2 | 8 |
| **E** | 41 | 0.25-16 | 2 | 8 |
| **Total** | 203 | 0.25-32 | 2 | 8 |

**Table	S4. The MIC distribution of VRC in each center.**

| **Center** | **N** | **MIC range** | **MIC₅₀** | **MIC₉₀** |
| --- | --- | --- | --- | --- |
| **A** | 38 | 0.008-0.125 | 0.016 | 0.125 |
| **B** | 41 | 0.008-0.25 | 0.016 | 0.25 |
| **C** | 33 | 0.008-0.125 | 0.016 | 0.125 |
| **D** | 36 | 0.008-0.5 | 0.016 | 0.25 |
| **E** | 52 | 0.008-0.125 | 0.016 | 0.125 |
| **Total** | 200 | 0.008-0.5 | 0.016 | 0.125 |

**Table S5. The MIC distribution of ISA in each center.**

| **Center** | **N** | **MIC range** | **MIC₅₀** | **MIC₉₀** |
| --- | --- | --- | --- | --- |
| **A** | 37 | 0.002-0.06 | 0.015 | 0.06 |
| **B** | 35 | 0.002-0.06 | 0.015 | 0.06 |
| **C** | 36 | 0.002-0.125 | 0.015 | 0.125 |
| **D** | 39 | 0.002-0.25 | 0.015 | 0.125 |
| **E** | 49 | 0.002-0.125 | 0.015 | 0.125 |
| **Total** | 196 | 0.002-0.25 | 0.015 | 0.125 |

**Table S6. The MIC distribution of ITC in each center.**

| **Center** | **N** | **MIC range** | **MIC₅₀** | **MIC₉₀** |
| --- | --- | --- | --- | --- |
| **A** | 38 | 0.008-0.06 | 0.016 | 0.06 |
| **B** | 36 | 0.008-0.125 | 0.03 | 0.125 |
| **C** | 35 | 0.008-0.125 | 0.016 | 0.125 |
| **D** | 36 | 0.008-0.5 | 0.016 | 0.25 |
| **E** | 44 | 0.008-0.5 | 0.03 | 0.5 |
| **Total** | 189 | 0.008-0.5 | 0.03 | 0.25 |

**Table S7. The MIC distribution of POS in each center.**

| **Center** | **N** | **MIC range** | **MIC₅₀** | **MIC₉₀** |
| --- | --- | --- | --- | --- |
| **A** | 38 | 0.008-0.125 | 0.03 | 0.125 |
| **B** | 35 | 0.008-0.125 | 0.016 | 0.125 |
| **C** | 37 | 0.008-0.125 | 0.016 | 0.125 |
| **D** | 39 | 0.008-0.25 | 0.016 | 0.25 |
| **E** | 55 | 0.008-0.125 | 0.06 | 0.125 |
| **Total** | 204 | 0.008-0.25 | 0.016 | 0.125 |
